# Alcohol oxidation as alternative anode reactions paired with (photo)electrochemical fuel production reactions

**DOI:** 10.1038/s41467-020-18461-1

**Published:** 2020-09-14

**Authors:** Michael T. Bender, Xin Yuan, Kyoung-Shin Choi

**Affiliations:** Department of Chemistry, University of Wisconsin-Madison, Madison, WI 53706 USA

**Keywords:** Energy, Materials for energy and catalysis

## Abstract

(Photo)electrochemical cells that produce fuels have often relied on water oxidation to complete the redox cycle. Here, the authors discuss alcohol oxidation as an alternative reaction and consider general mechanistic features of oxidation electrocatalysts.

## Electrochemical and photoelectrochemical cells for fuel production

The need to develop renewable and environmentally benign pathways to achieve fuel production is of increasing urgency. Aqueous reductive (photo)electrochemical reactions have received a great deal of attention as promising avenues through which this can be achieved. These reactions include water reduction to H_2_ gas, CO_2_ reduction to carbon fuels, and N_2_ reduction to NH_3_^1,2^. (Photo)electrochemical fuel production offers several attractive features including the ability to be conducted at ambient temperature and pressure, the use of water as the hydrogen source, and the ability to be driven by renewable energy sources (either directly by sunlight for photoelectrochemical reactions or by renewably generated electricity for electrochemical reactions). Additionally, (photo)electrochemical reduction of N_2_ and CO_2_ are achieved without prereducing water to H_2_, eliminating steps involved in the storage, delivery, and use of H_2_.

In (photo)electrochemical cells, production of the desired fuel at the cathode must be paired with an oxidation reaction at the anode. Traditionally, water oxidation through the oxygen evolution reaction (OER) has been used as this anode reaction. This choice has the advantage of being simple, as it does not necessitate the addition of any other species, and the O_2_ produced is environmentally benign. OER, however, is kinetically slow and requires a large overpotential. This results in an increase in the operating voltage of the cell (when the cell current is fixed) or a decrease in the rate of fuel production (when the input voltage is fixed). Additionally, while O_2_ is benign, it is not a highly valued product. As such, in recent years attention has been directed to finding a more attractive oxidative reaction that forms a value-added product that can be paired with reduction reactions of interest.

## Alcohol oxidation in biomass conversion as an alternative anode reaction

Features that make a reaction an attractive alternative to OER include: (1) being thermodynamically or kinetically more favorable than OER, (2) production of a valuable product, (3) production of H^+^ to maintain the overall solution pH (as all the aforementioned fuel production reactions consume H^+^), and (4) abundance of the reactant and high demand for the product. Regarding this last feature, we note that it is unlikely that the demand for any product or products produced at the anode will be as high as the demand for the fuels produced at the cathode. As such, it is unlikely that OER will ever be completely replaced by alternative anode reactions, meaning continued effort in improving the OER kinetics will be necessary. Developing more efficient and useful anode reactions, however, would also certainly help in increasing the commercial viability and efficacy of (photo)electrochemical fuel production cells.

A variety of reactions have been investigated in recent years as potential alternative anodic reactions that can be paired with fuel production, including oxidation of Cl^−^ to Cl_2_ or HClO, oxidation of water to H_2_O_2_, and oxidation of various organic molecules to molecules of greater value^3–9^. Among these reactions, oxidative valorization of biomass derived intermediates to produce commodity chemicals is of particular interest because it possess all of the aforementioned features required to be a promising alternative anode reaction. For example, electrochemical and photoelectrochemical H_2_ production has successfully been coupled with the oxidation of 5-hydroxymethylfurfural (HMF), a key intermediate produced from cellulosic biomass, to 2,5-furandicarboxylic acid (FDCA), a potential replacement for the terephthalic acid used in polyethylene terephthalate (PET) plastics^5^. Other examples include oxidation of glucose and glycerol, which can be produced abundantly from cellulosic biomass conversion and biodiesel production, respectively, to more value-added products such as glucaric acid and formic acid^6,7^. We note that all the above reactions involve alcohol and aldehyde oxidation to their corresponding carboxylic acids. Thus, these reactions can be generalized as the use of alcohol oxidation reaction (AOR) as an alternative anode reaction, a topic we will explore further below.

Because AOR as an alternative anode reaction will be performed in water under anodic bias, it must outcompete OER to achieve a high Faradaic efficiency (FE). Thus, it is reasonable to expect that the optimal catalytic electrodes for AOR would be those that are poorly catalytic for OER. Surprisingly, however, most of the non-noble metal-based electrocatalysts reported to achieve high FEs for these reactions are the same ones known to be good OER catalysts (e.g., Ni- and Co-based oxides, hydroxides, and oxyhydroxides)^6-9^.

When comparing the formulas of reactants and products for these two reactions (H_2_O and O_2_ for OER and RCH_2_OH and RCOOH for AOR), the reactant commonly loses hydrogen while gaining oxygen, meaning both reactions involve dehydrogenation and oxygen insertion. Thus, it is possible that OER and AOR share similar mechanistic steps for dehydrogenation and oxygen insertion, and this may be why the same catalysts are good at catalyzing both OER and AOR. Alternatively, if OER and AOR have significantly different mechanistic steps for dehydrogenation and oxygen insertion, it would mean that these catalysts possess some features that benefit both OER and AOR. To examine which of these possibilities is the case, we compare the general mechanisms for OER and AOR next.

## Comparison of general OER and alcohol oxidation mechanisms

**Fig. 1:**
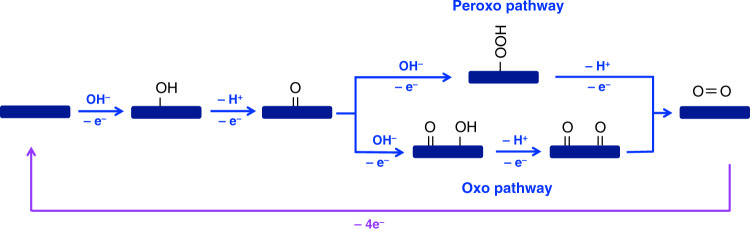
Schematic illustration of proposed OER mechanisms. Peroxo and oxo pathways for OER mechanisms on a transition-metal oxide/oxyhydroxide/hydroxide electrode surface in a basic solution are illustrated. The –e^−^ in blue indicates electrons transferred from hydroxide to the catalyst while –e^−^ in purple indicates electrons transferred from the catalyst to the back contact and eventually to the cathode.

The mechanism of OER has received extensive study; however, it is still the subject of some debate. The basic steps involved in OER are shown in Fig. 1. They are composed of the removal of protons from two hydroxide ions (or two water molecules if in low pH conditions) and the coupling of their oxygen atoms^10^. The oxygen coupling is proposed to be achieved either through formation of a peroxide intermediate on the catalyst surface (Fig. 1, Peroxo pathway) or through the coupling of two adjacent metal oxo species (Fig. 1, Oxo pathway)^10^. Both pathways involve formation of oxo species on the catalyst before the formation of O–O bonds.

**Fig. 2:**
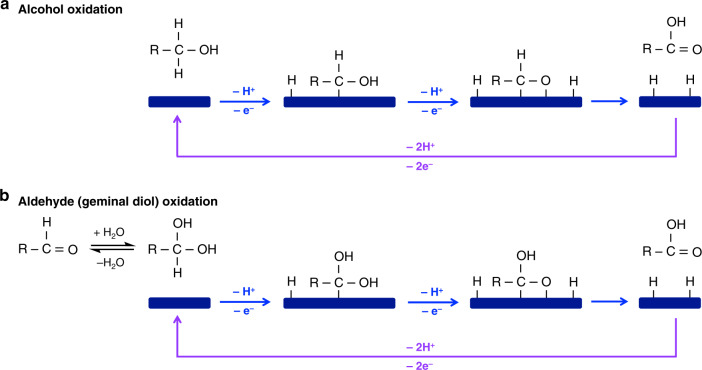
Schematic illustration of proposed alcohol and aldehyde oxidation mechnaisms on MOOH. The –e^−^ and –H^+^ in blue indicate those transferred from the organic molecule to MOOH, while the –e^−^ and –H^+^ in purple indicate those transferred from reduced MOOH (=M(OH)_2_) to the back contact and to a base in solution, respectively.

The mechanisms for electrochemical alcohol oxidation have received much less extensive study than those of OER, but the basic steps involved in AOR from the most established studies are shown in Fig. 2a. Like OER, AOR involves dehydrogenation (one hydrogen from the α-carbon and one hydrogen from the oxygen in the alcohol group). However, while the dehydrogenations in OER are through transfer of an acidic proton to a base in solution, the key dehydrogenation step in alcohol oxidation involves the removal of a nonacidic hydrogen at the α-carbon by cleaving the C–H bond^11,12^. Thus, the dehydrogenation in AOR must proceed through a different mechanistic process than does the dehydrogenation in OER. Studies by Fleischmann et al. in the early 1970’s using MOOH electrocatalysts (M = transition metals such as Ni and Co) reported that such reactions are mediated by the M(III)OOH/M(II)(OH)_2_ redox couple, with the rate limiting step being abstraction of the α-hydrogen by MOOH through a hydrogen atom transfer to form M(OH)_2_^11,13^. The active MOOH is then rapidly regenerated from the M(OH)_2_ due to the applied bias. Additionally, we note that under anodic bias and in aqueous solutions, MOOH is the catalytically active surface layer for most non-noble transition-metal-based catalytic anodes^14^. Thus, this proposed mechanism is likely applicable to a broad range of catalysts even if their bulk compositions are not MOOH.

The steps involved in oxygen insertion in OER and AOR are also quite different. While the formation of the O–O bond in OER involves the formation of oxo- and/or peroxo-species on the catalyst surface, the increase in the number of oxygens when aldehyde is oxidized to carboxylic acid is caused by the formation of geminal diol in solution (Fig. 2b)^15^, which is non-electrochemical and does not involve the adsorption of oxygen species to the catalyst surface. Once the geminal diol is formed, the oxidation of that geminal diol to carboxylic acid has the same dehydrogenation steps as the oxidation of alcohol to aldehyde. Thus, neither the dehydrogenation nor the oxygen insertion steps are the same for OER and AOR. This means that the reason why MOOH is good for both OER and AOR is because it possesses features beneficial to catalyzing both of the different sets of mechanistic steps involved in OER and AOR. These features are likely related to the ability of M in MOOH to cycle through multiple oxidation states and easily adapt its local structure when accepting hydrogen atoms for AOR or forming oxo- and peroxo-species for OER.

The reason why MOOH can achieve a high FE for AOR despite its ability to catalyze both AOR and OER is because MOOH uses different oxidation states of M for these reactions. As mentioned above, the dehydrogenation in AOR can be mediated by the M(III)OOH/M(II)(OH)_2_ couple, meaning that M(III)OOH has enough oxidation potential to perform alcohol oxidation. OER, on the contrary, requires further oxidation of the M(III) in MOOH to higher oxidation states^16^. Thus, when a potential is chosen to keep M(III) as the major species on the MOOH surface, AOR can be achieved with a high FE.

## Future direction to develop more efficient electrocatalysts for alcohol oxidation

We note that the AOR mechanisms used in the above mechanistic comparison are solely based on the earlier work on alcohol oxidation by Fleischmann et al.^11,13^. If the dehydrogenation of alcohol oxidation is achieved only through the hydrogen atom transfer mediated by the M(III)OOH/M(II)(OH)_2_ couple, the rate of alcohol oxidation on MOOH should be potential independent because the chemical oxidation of alcohol by M(III)OOH is the rate-determining step. However, a few studies including our recent study show that there is another mechanism for AOR that is enabled at potentials more positive than the potential to oxidize M(OH)_2_ to MOOH^8,17^. In this new mechanism, the rate of AOR appears to be potential dependent. This is advantageous because it may allow the rate of alcohol oxidation to be increased beyond what is achievable through the potential-independent oxidation using the M(III)OOH/M(II)(OH)_2_ couple. However, in the potential region that enables this alternative AOR mechanism, OER can also be turned on. Thus, further studies that elucidate the dehydrogenation steps involved in this new mechanism are important. Through these studies, strategies to enhance AOR over OER can be developed. These strategies will likely include optimizing the catalyst surface to promote alcohol adsorption and finding ways to promote the use of active sites for alcohol dehydrogenation over the formation of the oxo- and peroxo-species needed for OER. A better understanding of the two different AOR mechanisms will also allow for the tuning of the catalyst composition and structure as well as the operating condition to maximize the combined rate of AOR. This understanding will be beneficial not only for the use of AOR as an alternative anode reaction for (photo)electrochemical fuel production cells to produce commodity chemicals, but also for various aqueous electrochemical organic syntheses involving AOR.
